# How digital health risks recreating the isolation it aims to solve for people with serious mental illness

**DOI:** 10.3389/fmed.2025.1620156

**Published:** 2025-08-26

**Authors:** Waseem Jerjes, Daniel Harding, Arnoupe Jhass, Ashley Arif

**Affiliations:** ^1^Hammersmith and Fulham Primary Care Network, London, United Kingdom; ^2^Faculty of Medicine, Imperial College London, London, United Kingdom; ^3^Neurodiverse and Mental Health Advocate, London, United Kingdom

**Keywords:** serious mental illness, digital exclusion, primary care, health inequalities, relational continuity

## Introduction

People with serious mental illness (SMI), including schizophrenia, bipolar disorder, and other psychotic illness, continue to have disproportionately poor physical health outcomes in the UK. They have a reduced life expectancy of between 15 and 20 years on average compared to the general population, largely as a result of preventable physical illnesses such as cardiovascular disease, diabetes, and cancer (1, 2). Despite national policies of parity of esteem, only 30–50% of those with SMI receive preventive healthcare interventions such as cancer screening, immunisations, and annual health checks (2, 3).

The rapid growth of digital health services throughout and after the COVID-19 pandemic has introduced additional complexity to this landscape. The shift toward “total triage,” remote consultations, online booking systems, and patient portals has undoubtedly increased efficiency and access for many. However, it has also deepened digital inequalities—particularly for individuals with SMI, who are more likely to be digitally excluded due to low digital literacy, poverty, cognitive impairment, and mistrust of technology (4, 5). A national survey undertaken by the Mental Health Foundation found that adults with pre-existing mental health problems were among the least likely to use online health services, finding lack of confidence, access, and personalization to be underlying barriers (6).

This opinion article argues that, despite its promise, digital transformation in mental healthcare risks perpetuating the exclusionary logics of institutional care. Where once people with SMI were physically separated from society in asylums, now they may be digitally excluded—incapable of navigating systems, receiving care in a timely manner, or having their needs heard. Through the prism of primary care, which for many with SMI is still the most consistent point of contact, this paper explores how automation and depersonalization in digital service delivery can echo historical pathways of disconnection. It calls for a reconsideration of digital health—one where human connection, therapeutic continuity, and equitable access are foregrounded as fundamental pillars, not an afterthought. This opinion paper acknowledges the limitation of relying primarily on secondary evidence. To enhance empirical depth, qualitative insights from advocacy experiences are integrated to illustrate real-world dimensions of digital exclusion.

Key ethical concepts underpinning this viewpoint require clarification. “Human care” refers explicitly to an ethical orientation characterized by empathetic responsiveness, emotional sensitivity, and personalized interaction that prioritizes the specific psychological and emotional needs of the individual patient (7). This conceptualization draws from care ethics, notably Tronto's framework, which emphasizes attentiveness, responsibility, responsiveness, and relational engagement (8). The term “relationship” in this context describes sustained therapeutic interactions marked by trust, continuity, emotional safety, and mutual understanding—integral to mental health recovery and dignity-based care, as articulated by Nordenfelt (9).

### A historical parallel

For much of the 19th and early 20th centuries, people with SMI in the UK were removed from society and institutionalized in asylums—environments often justified as protective or therapeutic, but which came to foster social exclusion, dependence, and loss of control (10). These institutions, in offering shelter, also created rigid regimes in which patients had no autonomy, were stripped of community connections, and were rendered passive recipients of care. The asylum regime was characterized by hierarchical control, relational discontinuity, and impersonality—a system that, over time, came to embody marginalization in the name of care.

Although the closure of asylums and the evolution of community mental health care were significant strides forward, there is growing concern that aspects of modern digital health care can recreate some of the exclusionary patterns of the past. In today's digital-first systems—and particularly in the context of primary care triage—patients with SMI often find themselves excluded once more, not behind locked doors but behind password-protected portals, templated forms, and impersonal interfaces (5).

Individuals with complicated mental health needs are being funneled into asynchronous, algorithm-led pathways with little room for therapeutic contact or clinical judgment (11). A person seeking assistance may be requested to complete an online form, to subsequently receive a standardized reply, a referral to a different portal, or a telephone call from a healthcare worker (unknown to the patient) sometime afterward. The promise of access is technically fulfilled—but the quality of care is compromised. In some cases, individuals report being “referred to nowhere,” with no indication whether their message was received or understood.

Recent Care Quality Commission (CQC) reports have highlighted how digital systems can inadvertently perpetuate the disconnection already felt by vulnerable patients from health services, particularly where such systems are not relationally grounded or default to one-size-fits-all automation (12). While technology can be efficient, it can also bypass human contact—replicating, in new form, the very same dynamics of invisibility and powerlessness that were features of institutional psychiatry.

### How digital systems unintentionally diminish voice and agency

Online consultation systems are now ubiquitous across UK primary care, offering asynchronous appointment request, advice, or care initiation pathways. While such systems improve convenience and accessibility for some, they also introduce new barriers to communication—particularly for those with SMI (13). These systems rely heavily on patients being able to articulate their concerns in a coherent, legible, and clear way within restricted text boxes. Yet for individuals experiencing paranoia, cognitive disorganization, or flattened affect, the capacity for self-advocacy through the written medium can be severely impaired (14).

However, experiences of digital exclusion vary widely within the SMI population, influenced by specific diagnoses, cognitive abilities, socioeconomic factors, and cultural backgrounds. For example, individuals with paranoid schizophrenia might experience heightened anxiety around digital surveillance, whereas those with bipolar disorder may struggle particularly during depressive episodes to maintain sustained engagement with digital systems.

Having to navigate dropdown menus or construct coherent symptom narratives is, in and of itself, a barrier to access. For someone in psychosis or in the throes of a depressive episode, completing an online form may not only be difficult—it may be intrusive, even threatening. A recent report from Mind highlighted that many individuals with SMI actively avoid digital interactions for fear of being watched or misread, with some reporting online forms as making them feel depersonalized and ignored (15). The risk is not just disengagement, but silent decline.

Also, the majority of the e-consultation systems used in primary care lack advanced triage pathways for mental health. Suicidality, paranoia, or medication non-adherence may be buried in free text or inappropriately routed, with responses that are late or inappropriate (11). The systems are designed for clarity and rapidity—not for the subtle, often fragmented expressions of psychological distress that characterize many SMI presentations. What is lost in such a model is relational intelligence—the clinical instinct that is gleaned from tone, facial expression, pacing, and body language. In face-to-face or even telephone consultations, such cues often help clinicians to pick up on risk, tailor their communication, and build rapport. By comparison, a character-limited online submission strips away this clinical texture, reducing a complex human narrative to a digital snapshot.

As a result, people with SMI may not only receive slower, but also shallower care—less responsive, less human, and ultimately less safe. What is streamlined at the system level can, for the individual, be alienating or feel hopeless—leading to mounting mistrust and disengagement from health services.

### The psychological risks of digital-only mental health models

Digital systems are likely to be designed for efficiency—automating triage, standardizing communication, and accelerating access to care pathways. For individuals with SMI, however, the psychological experience of interacting with such systems can be profoundly negative. A chatbot interface or impersonal e-consult form, harmless as it may appear to others, can be perceived as robotic, invalidating, or even persecutory for an individual who is psychotic or severely depressed. The lack of human responsiveness can reinforce pre-existing persecutory, abandonment, or worthlessness beliefs (14).

For those who have experienced institutionalization or disconnection from services in the past, digital-only pathways risk re-opening old wounds. The system may appear to be “listening,” but its silence—or automated response—can be indistinguishable from rejection or abandonment. For some, patients report feeling more like data points than humans. Such reactions are not merely emotional reactions; they are guided by past experiences of marginalizing systems, frequently beginning in adolescence or young adulthood (15, 16).

This psychological burden is juxtaposed against the NHS Long Term Plan vision of personalized care, continuity, and improved access to mental healthcare (10, 11). While the plan supports digital innovation, it also acknowledges the need to reduce inequality and ensure inclusivity of services. Yet, there is a gap between policy intent and daily reality. As digital-by-default becomes standard, those with SMI are at risk of being pushed to the periphery—not by active exclusion, but by incremental erosion of significant contact.

Evidence from The King's Fund and The Health Foundation also shows that digital exclusion is strongly linked with loneliness, disengagement, and poorer health outcomes (17). If the sole entrance to care is one that is felt to be strange or menacing, patients will be less inclined to go through it. To be truly accessible, mental health services cannot simply open the door—they have to extend a human welcome.

### Recognizing diversity within serious mental illness

Acknowledging diversity within the SMI population is crucial to designing effective digital mental health interventions. Individuals with serious mental illness constitute a heterogeneous group, differing significantly in symptom presentation, cognitive function, emotional resilience, digital literacy, socioeconomic status, and cultural contexts (18). For instance, people experiencing psychosis may require interfaces that minimize feelings of surveillance or intrusion, while those experiencing severe depressive episodes might benefit from gentle, stepwise engagement strategies.

Socioeconomic differences also significantly influence digital access and engagement. Patients from lower socioeconomic backgrounds often experience compounded barriers, including limited device availability, unstable internet access, and lower baseline digital literacy, each requiring distinct interventions (14). Cultural background further shapes attitudes toward digital healthcare, influencing trust, perceived stigma, and willingness to engage.

Thus, digital health strategies must move beyond one-size-fits-all solutions, employing flexible, culturally sensitive, and personalized approaches tailored explicitly to the diverse realities of individuals within the SMI population.

### A primary care mandate

Primary care occupies a unique position in the UK healthcare system—not just as the first point of contact, but as an exemplar of long-term, relationship-based care (19). For policy proposals to be actionable within NHS primary care, they must explicitly consider existing operational constraints, institutional structures, and feasibility within current funding frameworks. The following strategies are proposed with clear implementation pathways tailored to the operational realities of NHS primary care settings. For people with SMI, who often have complex needs and fluctuating capacity to engage, this continuity is paramount. But as general practice embraces digital pathways at pace, it must negotiate a paradox: how to modernize systems without mechanizing relationships.

So far, much of the digital transformation has been focused on transactional efficiency—triaging demand, streamlining booking, and expanding remote access. These are necessary innovations, but insufficient when the patient journey is shaped as much by emotional safety and therapeutic rapport as clinical urgency. However, it would be overly simplistic to frame digital technology and human care as inherently oppositional. Evidence indicates that thoughtfully implemented digital interventions, such as video consultations and personalized messaging services, can significantly enhance relational continuity, improve patient engagement, and facilitate timely care delivery. If general practice is to take a lead on digital mental health care for people with SMI, it must create systems that invite patients in—not merely process them through.

### Bridging technology and relational care

Emerging research and NHS pilot studies illustrate how digital technology can positively impact the relational dimensions of healthcare (20). For instance, digital platforms enabling video consultations have allowed continuity of therapeutic relationships, particularly important for patients experiencing severe anxiety or social withdrawal. Similarly, text-based interactions, managed sensitively, can support those who find face-to-face interactions overwhelming or anxiety-inducing, thus enhancing their ability to express concerns more freely.

Successful case examples include NHS digital initiatives where patient-clinician relationships were notably strengthened through virtual follow-up appointments, thereby providing emotional reassurance and maintaining a consistent therapeutic presence (21). These examples underline the potential for digital tools, if appropriately designed, to be integrated seamlessly into relational care models. Thus, rather than replacing human interaction, technology can serve as a meaningful adjunct to enhance relational quality and clinical effectiveness.

Digital triage systems, as presently configured, have a tendency to assume a level of self-advocacy and coherence that individuals with SMI are not able to sustain consistently. One answer is to re-design such systems to work as connectors rather than filters. Instead of screening out complexity, digital interfaces must be configured to sense vulnerability, cue relational responses, and fast-track continuity. This would require not only technical redesign but a philosophical shift in how practices respond to digital demand, in essence seeing it as an opportunity for meaningful contact.

Practices can utilize digital flags in their systems to alert staff to individuals with known mental health diagnoses, previous safeguarding concerns, or a history of disengagement. These alerts can then prompt proactive outreach—often by a known team member—who can make first contact through the patient's preferred method, whether that is telephone, text, or face-to-face.

This approach must be matched with investment in reshaping the workforce. While many practices have built their multidisciplinary teams, relatively few have created posts that bridge the digital and relational divide explicitly. The creation of digital inclusion or navigation posts could alter the landscape. Such individuals would not merely offer IT support but would be central to the mental health offer—navigating patients through online processes, booking appointments on their behalf, and being a human voice within an increasingly digitized system. Most critically, they could work alongside social prescribers, community mental health nurses, and mental health practitioners based in primary care networks, so that digital access became a part of a whole wellbeing plan and not a separate skillset.

Another essential strategy is in solidifying co-production as a design principle by default. Too often, systems are designed around assumptions regarding what patients need, rather than from direct contact with lived experience. Practices must co-collaborate with patients who have used, avoided, or struggled with digital systems, to understand precisely which features exclude or facilitate them. Co-design workshops—bringing together service users, clinicians, administrative staff, and developers—can yield actionable redesigns, from simplifying forms to offering alternative points of contact at every digital touchpoint. Additionally, such collaborations help restore agency for individuals with SMI, who commonly report feeling invisible within healthcare settings. By having input into the design of their care access, they regain a sense of control and visibility.

Another often overlooked but essential element is the role of reception and front-desk staff. In digitally enabled practices, these staff still deliver the essential human interface of general practice. Yet they are rarely trained to recognize digital exclusion or when frustration masks deeper disengagement. Practices must introduce formal training modules for administrative staff that allow them to recognize SMI-related access problems, communicate with flexibility, and escalate with appropriateness. This kind of mental health literacy must be a core competency, not a nice-to-have add-on.

Continuity of care must be reasserted as a digital design objective. In the era of complete triage and shared clinician rotas, patients are increasingly likely to be assigned to the next available clinician, regardless of past relationship. While understandable from an access perspective, this model erodes the longitudinal relationships that are most protective for those with SMI. Practices should be encouraged—through contractual levers and incentives—to prioritize continuity for individuals on serious mental illness registers. Digital booking systems should display preferred clinicians where possible, or at least allow patients to flag a continuity request. In addition, patients at risk of disengagement could be actively offered a named care coordinator or clinician who follows up on them across channels. Such personalization reduces the emotional and cognitive labor of having to reintroduce oneself repeatedly, a prevalent barrier to continued engagement.

At a system level, commissioning structures must look beyond access measures alone and begin to value qualitative measures of digital inclusion. Current targets emphasize response times and take-up rates, but rarely interrogate whether digital systems are running equitably across patient groups. Integrated Care Boards (ICBs) could commission digital inclusion audits of primary care networks to identify which populations are underrepresented in digital traffic, and why. Practices could be resourced to respond with tailored interventions—whether that's offering digital literacy sessions, expanding phone-based support, or creating hybrid consultation models for patients who fall between digital and face-to-face preferences.

Finally, national policy is urgently needed to grasp the complexity of digital transformation. Digital-first care has been hailed as an answer to access and sustainability. But for SMI groups, a different approach is required. NHS England could consider issuing guidance on digital mental health accessibility in primary care, as it has with the current accessible information standard. This would help to make reasonable adjustments—such as offering offline alternatives, longer appointment times, or low-stimulus digital spaces—a standard expectation and not reactive exceptions. Most importantly, this would convey that inclusion is a safety issue and not a service design preference.

### Operational and institutional feasibility of recommendations

The practical implementation of these recommendations is grounded in current NHS infrastructure and funding realities (22). For instance, the creation of dedicated “digital inclusion navigator” roles can be feasibly integrated into primary care teams through existing funding streams such as the Additional Roles Reimbursement Scheme (ARRS). These roles would align closely with current Social Prescribing Link Workers and Care Coordinators, leveraging existing budgets without requiring entirely new funding streams.

Training administrative staff in digital mental health literacy can be incorporated into annual mandatory training schedules already established in NHS practices. Primary Care Networks (PCNs) can lead this initiative, supported by established NHS England guidance and resources already available through NHS Digital and Health Education England (HEE).

Qualitative digital inclusion audits could be readily embedded into existing Integrated Care Board (ICB) contractual frameworks, complementing current performance metrics such as Quality and Outcomes Framework (QOF) targets and Digital First Primary Care initiatives. By explicitly including digital inclusion criteria in primary care contractual frameworks, feasibility is assured through existing NHS commissioning structures.

Lastly, developing national NHS England guidance for digital accessibility in mental health could follow precedent set by the Accessible Information Standard, making reasonable adjustments mandatory, clearly defined, and financially accounted for within existing practice-level NHS funding mechanisms (23).

## Discussion

The journey of digital transformation in healthcare has been one of acceleration—urgent, innovative, and often imperative (24, 25). But rushed progress, without reflection, risks perpetuating the exclusions it seeks to resolve. For those with SMI, these risks are not hypothetical; they are already happening in silence—cues overlooked, needs neglected, and interactions that feel more like transactions than care. As the NHS rebalances access, it must appreciate the difference between availability and accessibility, between digital presence and engaged participation.

We must be willing to ask other questions. Not whether digital health can reach more people, but whether it reaches the right people in the right way. Not whether it can replicate clinical tasks, but whether it can preserve clinical relationships. In mental health, where recovery often hinges on trust, predictability, and emotional safety, these are the distinctions that are central—not peripheral—to effectiveness.

Primary care, long valued for its continuity and cultural familiarity, is one of the few remaining clinical settings where therapeutic relationships are established over time rather than transactions (26). It is here that people with SMI often find solid ground, sometimes after years of fragmented or institutional care. If digital systems are to augment this ethos rather than undermine it, they must be designed with attentiveness—rather than automation—as the governing principle. Technology must learn to mirror the values of human practice, not just its procedures.

But what if the greatest risk of digital mental health systems is not exclusion, but misrecognition? A system can log a completed form, assign a triage score, and generate a care plan—and still fail to see the person behind the symptoms. For people with SMI, whose presentations of distress are unlikely to conform to tidy categorization, algorithmic processes risk flattening complex narratives into convenient templates. In the longer term, this can distort how clinicians see patients—and more worryingly, how patients come to see themselves: not as whole persons, but as clinical fragments scattered across interfaces.

This, then, begs a deeper ethical question: what is the humanity envisioned by our digital systems? When the ideal is efficiency, vulnerability is treated as friction to be engineered out. But a compassionate system must do the opposite—it must embrace friction. It must recognize that slowness, confusion, repetition, or hesitation are not obstacles to care but indicators of the very need for it. True innovation will not come from the elimination of human complexity, but from how we learn to remain present within it.

Moving ahead, digital technologies must be gauged not just by uptake rates or resolution times, but by how well they work for those least able to navigate them on their own. Non-use, avoidance, and digital silence must be handled as clinical signals, not system noise. Listening to absence—what is not said, not read, not submitted—will be among the most important diagnostic tools of the era of remote care (26, 27). Lastly, the fate of online mental health will be decided less by platform design than by posture. A posture that is relational, reflexive, and humble. Technology can widen reach, but only humans can reconstruct meaning. Our task is not to settle whether machines can care—it is to ensure that where they cannot, someone still does (28).

### Patient perspectives on digital exclusion: insights from advocacy

Empirical narratives drawn from advocacy experiences provide valuable insights into the lived realities of digital exclusion among people with serious mental illness (SMI). Patients frequently describe digital-first approaches, such as online consultations and e-forms, as impersonal and alienating, especially during periods of acute psychological distress. Advocacy examples highlight practical and emotional barriers, including difficulty expressing psychological distress via restrictive digital interfaces, anxieties about surveillance, and confusion from unclear navigation.

Advocates report that many individuals with SMI perceive digital systems as reinforcing existing feelings of invisibility and disconnection from health services, thereby heightening their sense of vulnerability. Moreover, the impersonal nature of automated triage systems is often experienced as invalidating, creating an additional psychological burden for individuals already dealing with profound mental health challenges. Such narratives emphasize the critical need for digital health services to prioritize flexibility, relational responsiveness, and empathetic interaction to ensure genuine accessibility.

Thus, incorporating these patient-centered insights strengthens the paper's empirical foundation, illustrating clearly the complexities digital exclusion poses to people with serious mental illness and highlighting crucial areas for practical improvement within digital health strategies.

## Conclusion

General practice can't subcontract its relational role to algorithms. Nor can it be left to bear the brunt of digital transformation without structural support. But what it can do—better than any other part of the health system—is listen, adapt, and personalize. With the right tools, training, and ethos, primary care can lead a quiet revolution: one in which technology does not supplant the human touch, but enhances it. For people with serious mental illness, this is not a luxury—it's the difference between access and absence, connection and collapse. Digital mental health must be built around this reality, with primary care as its staunchest advocate and most thoughtful steward.
